# Do-not-intubate status and COVID-19 mortality in patients admitted to Dutch non-ICU wards

**DOI:** 10.1007/s10096-021-04223-4

**Published:** 2021-03-12

**Authors:** Tjeerd van der Veer, Simone van der Sar-van der Brugge, Marthe S. Paats, Els van Nood, Ingrid C. de Backer, Joachim G. J. V. Aerts, Menno M. van der Eerden

**Affiliations:** 1 Department of Pulmonary Medicine, Erasmus MC, Rotterdam, the Netherlands; 2 Department of Pulmonary Medicine, Amphia Hospital, Breda, the Netherlands; 3 Department of Internal Medicine, Erasmus MC, Rotterdam, the Netherlands; 4 Department of Medical Microbiology and Infectious Diseases, Erasmus MC, Rotterdam, the Netherlands

**Keywords:** COVID-19, Mechanical ventilation, Elderly medicine, Respiratory intensive care, Viral respiratory infection, Advance care planning

## Abstract

Mortality from COVID-19 has been particularly high in elderly patients on mechanical ventilation. Treatment outcomes for patients with do-not-intubate (DNI) status are unknown. One hundred patients admitted to the non-ICU ward during the “first wave” were retrospectively analyzed. Mortality rate was 49% in patients with a DNI order. This subgroup was characterized by significantly higher age, more comorbidity, and care dependency. Mortality among DNI patients was three times higher than other patients, but not higher than some of the published mortality rates for elderly mechanically ventilated patients. Advanced care planning is essential in COVID-19 to assist patient autonomy and prevent non-beneficial medical interventions.

## Introduction

In-hospital mortality rates due to COVID-19 have primarily been determined by ICU mortality. In the first months of the pandemic, there was an average 42% risk of mortality when admitted to the ICU [1], while overall mortality of hospitalized patients was around 20% [2, 3]. Mortality is particularly high for mechanical ventilation at older age [1–3]. Some elderly and/or frail patients are not admitted to the ICU for mechanical ventilation and remain on the non-ICU ward for conservative treatment. In the Netherlands, advance directives such as a do-not-intubate (DNI) order are required to be discussed with the patient and/or relatives upon admission. To characterize a group of patients in whom a DNI status was chosen upon admission and to compare outcomes with patients without treatment restrictions, we analyzed 100 patients admitted to the non-ICU COVID-19 wards.

## Methods

The Erasmus MC is the Netherlands’ largest university hospital, and Amphia Hospital is a large teaching hospital. In this retrospective analysis, we reviewed the files of 100 patients (the first 50 patients per center) admitted to the dedicated non-ICU COVID-19 wards, with admission dates between 17 March and 23 April 2020. Cases of COVID-19 were all confirmed by real-time PCR of nasopharyngeal swabs. Of these 100 patients, patient characteristics, clinical signs and symptoms, comorbidities, treatment, and outcomes were compared between patients with and without a DNI order. Comorbidities were scored using Charlson comorbidity index (CCI) [4].

## Results

Of 100 patients, the average age was 68 years, body mass index (BMI) was 27, and 67% of the patients were male. Of these patients, 82% had at least one pre-existing comorbidity, with an average Charlson comorbidity index (CCI) of 4.2 points (see Table 1).

**Table 1:** Characteristics, symptoms, treatment and outcomes of patients admitted to the non-ICU COVID wards

Patient characteristics	DNI (*n*= 39)	Non-DNI (*n*=61)	Total (*n*= 100)
Age*	77.0 years ± 9.2 Range 61–94	62.2 years ± 13.2 Range 21–85	68.0 years ± 13.8 Range 21–94
BMI	26.7 ± 5.8	27.2 ± 4.6	27.0 ± 5.1
Male/female	67%/33%	67%/33%	67%/33%
Charlson comorbidity index*	6.2 ± 2.3	2.9 ± 2.2	4.2 ± 2.7
Any kind of chronic comorbidity*	97.4%	73.8%	82%
Living at home unassisted*	69.2%	96.7%	86%
Living with care at home*	18%	3.3%	9%
Living at care facility*	12.8%	0%	5%
Outcomes			
Mortality*	48.7% (19/39)	16.4% (10/61)	29% (29/100)
Admission until discharge (survivors), days	10.4 ± 5.8	10.1 ± 8.1	10.2 ± 7.5
Discharge to home vs. other care facility *	40%/60%	82.4%/17.6%	70.4%/29.6%
Admission until death (deceased), days†	6.7 ± 4.7	12.1 ± 7.2	8.6 ± 6.1
Symptom onset to admission, days*	4.7 ± 3.9	9.7 ± 4.8	7.8 ± 5.1
Symptoms and treatment at admission			
Dyspnea	69.2%	82.0%	77.0%
Temp ≥38.0	59.0%	73.8%	68.0%
Oxygen suppletion at admission	61.5%	54.1%	57.0%
Treatment with steroids	10.3%	14.8%	13.0%
Hydroxychloroquine	33.3%	45.9%	41.0%

*p< 0.01 for comparison between DNI and non-DNI groups. †*p*<0.05 for comparison between DNI and non-DNI groups. *p*-values calculated using *t*-test and chi-square test

For 39 patients (39%), a DNI order was noted in the patient file. In 29/39 patients (74%), the DNI status was chosen at admission after counselling by the attending physician. For 8/39 patients (21%), the DNI status had already been chosen previously. For two patients (5%), the DNI status was decided during hospital stay after confirmation of COVID-19 diagnosis because of expected poor prognosis of mechanical ventilation for COVID-19 pneumonia. The reported reasons to choose a DNI status were combined frailty, multimorbidity, and age (23/39, 59%); the patient’s explicit wish (5/39, 13%); severe neurologic comorbidity (3/39, 7.5%); active malignancy (2/39, 5%); cardiac comorbidity (2/39, 5%); pulmonary comorbidity (1/39, 3%); and severe cognitive impairment (3/39, 7.5%).

Patients with a DNI status were significantly older in comparison with patients without a DNI status, (77.0 vs. 62.2 years, *p*<0.01), had more comorbidities resulting in a significantly higher CCI (6.2 vs. 2.9 points, *p*<0.01), and lived significantly less often at home unassisted (69.2% vs. 96.7%, *p*<0.01) (see Table 1).

Overall mortality rate was 29%. Outcomes differed significantly between DNI and non-DNI status groups. Among patients with a DNI status, mortality was 48.7%. No DNI status patients were later transferred to the ICU. Among patients without a DNI status, mortality was 16.4%. Of this group, 16 patients (26.2%) were admitted to the ICU for mechanical ventilation when their condition deteriorated, of which 10/16 patients died (62.5%) (see Table 1).

Patient symptoms at admission were not significantly different, except for the reported number of days since onset of symptoms, which was significantly higher for the group without DNI status. Initial treatment did not differ significantly between groups. All patients were treated with oxygen suppletion by nasal cannula or non-rebreathing oxygen masks. During this period, no high-flow nasal oxygen devices were used because of concerns of COVID-19 spread through aerosolization. All patients received empiric treatment with broad spectrum antibiotics. A minority of cases received either steroids or hydroxychloroquine, with no significant differences for comparison between DNI and non-DNI groups. (Of note, these patients were admitted before the beneficiary effects of steroids were known [5] and before implementation of standard high prophylactic antithrombotic medication.)

## Discussion

Patients with severe COVID-19 are often transferred to the ICU for mechanical ventilation. However, in some circumstances, patients are not eligible for invasive mechanical ventilation, because of their own wishes or severe comorbidity and/or frailty. Mortality rates for this category of patients have not yet been published. Our retrospective study shows that mortality rates were very high for this category of patients (48.7%) and three times higher than patients without DNI status admitted to the same wards in the same period. It is also about two times higher than overall COVID-19 mortality in our hospitals during the same time period (circa 25%, unpublished data) and “first wave” mortality rates in the literature [2, 3]. Compared to the non-DNI group, patients with a DNI status had significantly higher age and had more comorbidity, which are known risk factors for COVID-19-related mortality. DNI patients were on average 77 years old with an average CCI score of 6.2, which indicates multiple comorbidities and an estimated 10-year survival of 0–2%. Mortality rates reported for invasively ventilated patients in this age group in Germany were even higher at 63% (70–79 years) and 72% (patients over 80 years), whereas mortality in non-invasively ventilated patients was 45% [2]. In another analysis of octogenarian patients, premorbid frailty was an important prognostic factor, and ICU treatment did not improve outcomes in fit nor frail patients [6]. Compared with these figures, there is no sign that mortality rates for our patients have been negatively impacted by the DNI policies.

Remarkably, only 5 patients were living at a nursing home prior to admission. In all those patients, a DNI status was ordered. Many nursing home residents with COVID-19 have been cared for in their facility and were not referred to the hospitals. Advanced care planning including patient’s views on resuscitation, intubation, and referral to hospitals are routine practice in Dutch nursing homes, allowing optimal attention to palliative care where indicated and prioritization of advanced medical care to the patients with greatest benefit. Although total capacity and logistics of ICU beds for COVID-19 patients became a topic of national interest during the weeks of peak incidence, there was no “black list” for triaging ICU access. In our cohort, ICU capacity was available for all eligible patients, and all DNI orders were chosen after counselling with patients and/or their relatives. In recently published guidance on advanced care planning in COVID-19, this practice has been recommended as standard care [7, 8].

In conclusion, during the peak of the COVID-19 outbreak in the Netherlands, mortality in the dedicated non-ICU COVID-19 wards of our centers was high, especially in patients who had a DNI status. This group of patients was characterized by old age, high burden of comorbidities, and assisted living or nursing home residency. Advanced care planning is essential in COVID-19 to assist patient autonomy and prevent non-beneficial medical interventions.

### Data Availability (data transparency)

The datasets generated during and/or analyzed during the current study are available from the corresponding author on reasonable request.

### Code availability

Not applicable
